# Myeloma Cast Nephropathy and COVID-19: A Case Report of Multiple Myeloma Presenting as Acute Kidney Injury in the Setting of COVID-19

**DOI:** 10.7759/cureus.14461

**Published:** 2021-04-13

**Authors:** Waqas Memon, Karishma Popli, Ayesha Akram, Sindhura Bobba, Selvaraj Muthusamy

**Affiliations:** 1 Internal Medicine/Nephrology, Virginia Commonwealth University, Richmond, USA; 2 Medicine, Virginia Commonwealth University School of Medicine, Richmond, USA; 3 Internal Medicine, Combined Military Hospital, Rawalpindi, PAK; 4 Internal Medicine, Rawalpindi Medical University, Rawalpindi, PAK; 5 Transplant Nephrology, Hunter Holmes McGuire VA Medical Center, Virginia Commonwealth University, Richmond, USA; 6 Renal Pathology, Virginia Commonwealth University, Richmond, USA

**Keywords:** multiple myeloma, diagnosis of multiple myeloma, nephrotic syndrome, dialysis, covid 19

## Abstract

A 64-year-old African American male presented to the emergency department with subacute low back pain for two weeks and decreased urine output. He was found to have a potassium level of 9.2 mmol/L and was uremic with a creatinine level of 28.5 mg/dL and blood urea nitrogen (BUN) level of 201 mg/dL. He also tested positive for COVID-19. He was then started on continuous renal replacement therapy (CRRT). His urinalysis showed more than 500 mg/dL of protein. A workup for multiple myeloma was also conducted, and urine protein electrophoresis test was positive for free lambda light chains with a level of 17,700 mg/L and free kappa light chains with a level of 88.30 mg/L with a kappa:lambda free light chain ratio of 0.005. Additionally, serum Bence Jones protein level was elevated at 240 mg/dL, and serum beta-2 microglobulin level was elevated at 31.41 mg/L. An immunoglobulin (Ig) panel also showed low levels of IgG, IgA, and IgM. Kidney biopsy for this patient showed definite cast nephropathy and minimal chronic changes, with only one of over 20 glomeruli sclerosed and minimal interstitial deposits. The patient was started on chemotherapy with cyclophosphamide, bortezomib, and dexamethasone (CyBorD).

## Introduction

Monoclonal gammopathy is known to be a significant cause of renal disease, most notably light-chain cast nephropathy seen in multiple myeloma (MM) [1]. MM accounts for approximately 17% of all hematological malignancies and typically requires evidence of end-organ damage attributable to neoplastic plasma cell proliferation including hypercalcemia, renal failure, anemia, and osteolytic bone lesions [2,3]. Approximately 20-50% of patients diagnosed with MM initially present with acute kidney injury (AKI), with up to 12% requiring hemodialysis [4,5]. Studies have shown that patients with a severe AKI complicating MM, especially upon diagnosis, have worse outcomes, with a median survival of less than one year compared to three years for patients with moderate renal impairment in the setting of MM [6,7]. Increased deposition of nephrotoxic immunoglobulin (Ig) free light chains obstructing the distal lumen and leading to myeloma cast nephropathy is responsible for approximately 70% of dialysis-dependent renal failure. Treatment focusing on rapid reduction of these proteins as well as reversal of renal impairment could potentially be beneficial [7-9].

There is limited information regarding the characteristics of MM being diagnosed in the setting of a concurrent coronavirus disease 2019 (COVID-19) infection or regarding the prognostic factors and clinical outcomes of patients hospitalized for MM and COVID-19. COVID-19 infection has been independently associated with AKI [10], but concurrent newly diagnosed MM upon hospitalization with an AKI in the setting of COVID-19 has not been reported in the literature. Patients with MM are more susceptible to infections due to humoral and cellular immune dysfunction, and this specific patient population has been shown to have worse clinical outcomes when hospitalized for COVID-19 infections. A recent retrospective study in Spain found that inpatient mortality for patients with COVID-19 and MM was greater compared to patients without MM and that patients with comorbid renal disease at the time of hospitalization had significantly worse clinical outcomes and greater mortality rates [11]. Another case study in Qatar showed similar results with worse prognosis and higher mortality rates in patients newly diagnosed with MM undergoing treatment who were subsequently infected with COVID-19. Patients with renal failure requiring hemodialysis during their hospitalization typically had the highest mortality rates [12]. Another study by the International Myeloma Society found that the independent predictors associated with adverse outcomes in patients with MM and COVID-19 infection included renal disease, age, high-risk MM, and suboptimal MM control [12].

 We describe a case of a 64-year-old male with subacute low back pain, which did not improve with medical management, who presented with an AKI in the setting of a concurrent COVID-19 infection and later developed myeloma cast nephropathy and was diagnosed to have MM.

## Case presentation

A 64-year-old African American male with a history of hypertension, hyperlipidemia, type 2 diabetes mellitus, and hyperplasia of the prostate presented to the emergency department with subacute low back pain for two weeks, which did not improve with conservative medical management, and symptoms of weight loss, anorexia, ageusia, nausea without emesis, chills, fatigue, malaise, and weakness. The patient endorsed taking ibuprofen 400 mg every six hours over the past two weeks without relief of symptoms and noted decreased urine output without dysuria or hematuria. The patient was admitted to the intensive care unit (ICU) due to renal failure with hyperkalemia with a potassium level of 9.2 mmol/L, uremia with a creatinine level of 28.5 mg/dL, blood urea nitrogen (BUN) level of 201 mg/dL, and glomerular filtration rate (GFR) of 2 mL/min/1.73m^2^, and anion gap metabolic acidosis with a carbon dioxide level of 8mmol/L, blood pH of 7.14, and anion gap of 22 mEq/L. Upon admission, he also tested positive for COVID-19, and later radiographic chest imaging revealed right middle lobe infiltrate.

The patient was initially diagnosed with a stage 3 non-oliguric AKI, with hyperkalemia and uremia thought to be due to normotensive ischemic renal failure due to his multiple co-morbidities, concurrent COVID-19 infection, and medication usage including NSAIDs (nonsteroidal anti-inflammatory drugs) and angiotensin-converting enzyme (ACE) inhibitors. The patient received medical management for hyperkalemia; however, later he became anuric and was started on hemodialysis via trialysis line. However, he experienced intermittent hypotensive episodes and two seizure episodes on hemodialysis despite receiving mannitol thought to be due to dialysis disequilibrium syndrome. He was then started on continuous renal replacement therapy (CRRT) and developed a mixed metabolic and respiratory alkalosis with pH of 7.49, an arterial partial pressure of carbon dioxide of 36 mmHg, and an arterial partial pressure of oxygen of 82 mmHg.

Notable labs for this patient upon admission included leukocytosis with a white blood cell count of 15.1 K/mL and anemia with a hemoglobin level of 8.9 g/dL. The patient continued to have back pain throughout his ICU stay, one episode of melena, and intermittent episodes of altered mental status. His hemoglobin level dropped to a trough of 5.9 g/dL on the second day of admission, and his iron panel was consistent with anemia of chronic disease with a hypoproliferative absolute reticulocyte count of 0.01 cells x 10^6^/mL. His urinalysis showed more than 500 mg/dL of protein and a urine protein-to-creatinine ratio of 9:1, which is in the nephrotic range. The patient also had an active urine sediment with 273 WBCs/hpf and 4,532 RBCs/hpf, which raised concern for rapidly progressive glomerulonephritis since his creatinine prior to this admission was previously normal. A full hepatitis and autoimmune antibody panel did not reveal any abnormalities, ruling out other possible causes for his nephrotic syndrome and AKI.

A workup for MM was also conducted given his symptoms and clinical findings of subacute back pain, anemia of chronic disease, discrepant urine protein-to-creatinine ratio, and microalbumin-to-creatinine ratio, and acute renal failure. A urine protein electrophoresis test was positive, and serum protein electrophoresis revealed hypoalbuminemia, with free lambda light chains with a level of 17,700 mg/L and free kappa light chains with a level of 88.30 mg/L, with a kappa:lambda free light chain ratio of 0.005. Additionally, serum Bence Jones protein level was elevated at 240.0 mg/dL, and serum beta-2 microglobulin level was elevated at 31.41mg/L. An Ig panel also showed low levels of IgG, IgA, and IgM. Bone marrow biopsy revealed sheets of plasma cells (approximately 80% of total cells). Many of the plasma cells were positive for CD56. Kappa and lambda in situ hybridization showed lambda light chain restriction in the plasma cells.

**Figure 1:**
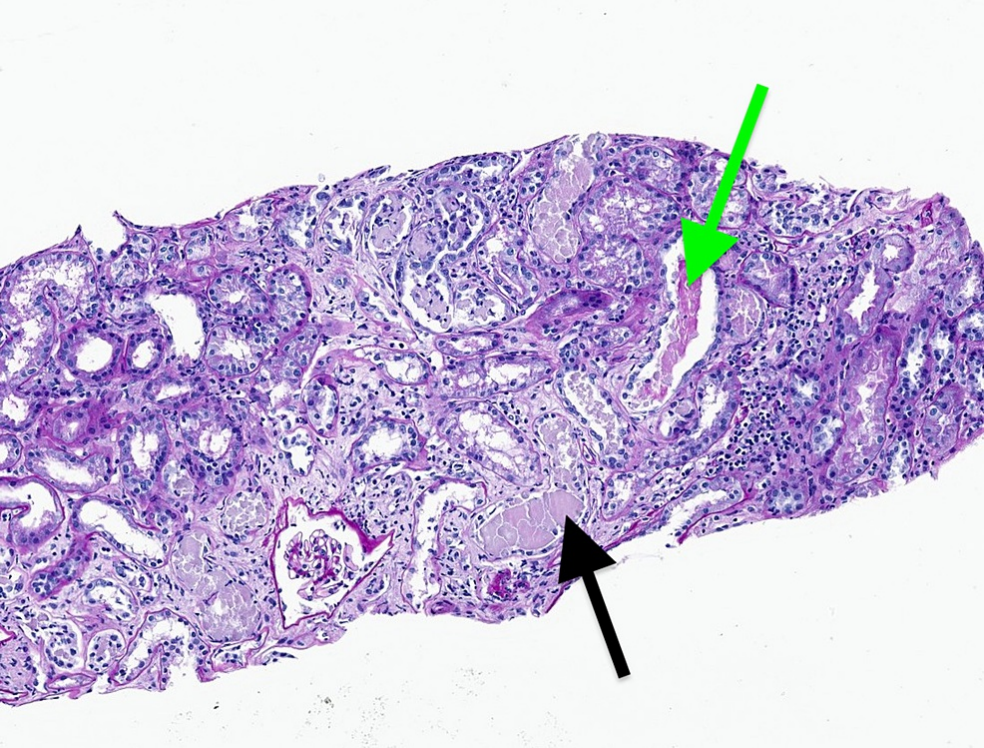
Image showing atypical tubular casts demonstrating pale staining with PAS, fractured eosinophilic casts (black arrow), and adjacent inflammatory infiltrate (green arrow), all suggestive of ongoing light chain cast nephropathy. PAS, Periodic acid–Schiff

**Figure 2:**
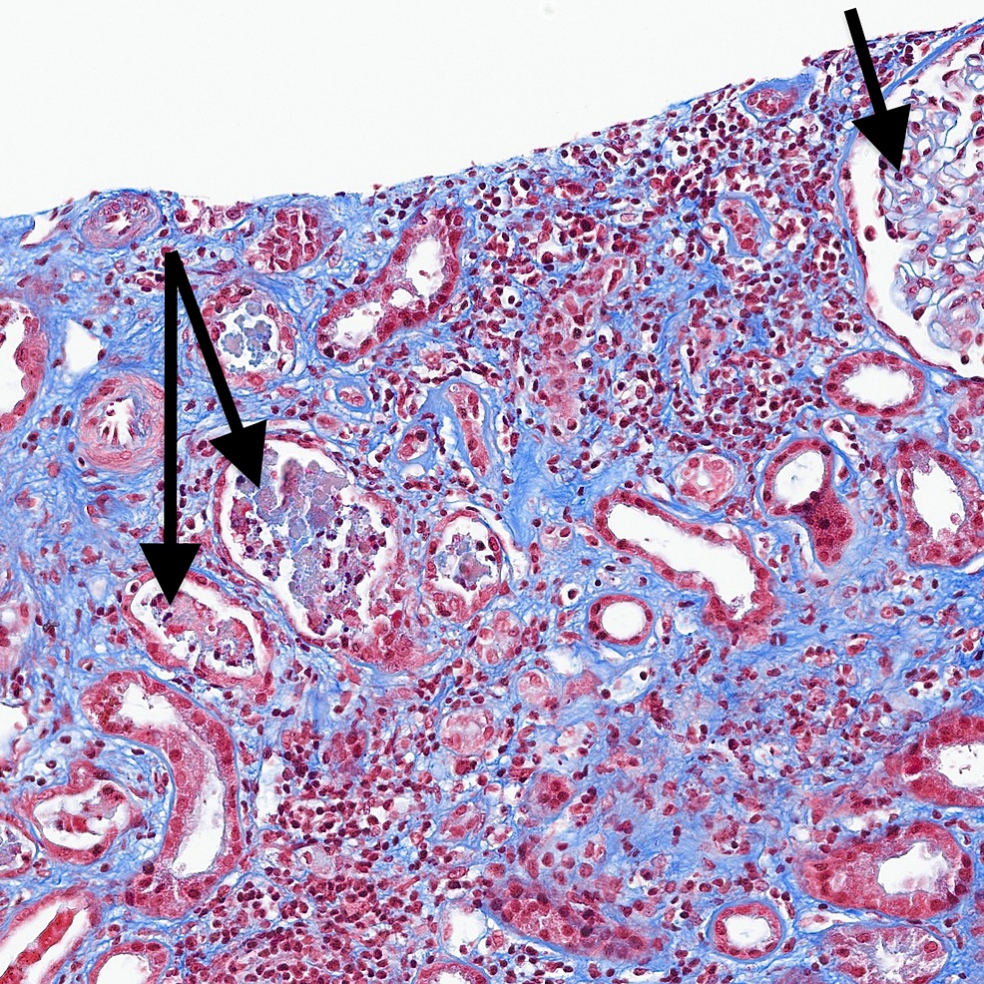
Trichrome stain showing atypical casts with adjacent inflammatory cell infiltrate (black arrows).

Kidney biopsy of this patient showed light chain cast nephropathy with mild interstitial inflammation and fairly extensive interstitial fibrosis (Figures 1, 2). Mild sclerosis and interstitial fibrosis most likely attributable to the patient's chronic co-morbidities. The patient was started on chemotherapy with cyclophosphamide, bortezomib, and dexamethasone (CyBorD). The patient’s AKI secondary to cast nephropathy was treated with plasmapheresis for six cycles to help decrease light chains and improve kidney recovery. A bone marrow biopsy using hematoxylin and eosin stain showing bone marrow plasma cells ≥ 60%. The patient received plasmapheresis and is currently receiving hemodialysis on Tuesdays, Thursdays, and Saturdays. He is currently being evaluated for autologous hematopoietic stem cell transplantation.

## Discussion

MM is a lymphoproliferative disorder characterized by monoclonal plasma cell proliferation and the production of monoclonal Igs. It should be suspected in elderly patients with any combination of bone pain (often in the back and ribs due to lytic lesions) with hypercalcemia, normocytic anemia, elevated gamma gap, or acute renal failure [5]. Acute renal failure in MM is caused by light chain cast nephropathy. Free light chains (Bence Jones proteins) are filtered by the glomerulus, and when levels exceed reabsorptive capacity, light chains precipitate with Tamm-Horsfall proteins, forming casts that cause tubular obstruction and epithelial injury [5].

On light microscopy (LM), atrophic tubules containing large, waxy, eosinophilic casts is a definitive diagnosis that AKI may have been caused by MM rather than COVID-19 in a patient with concurrent MM and COVID-19. Free light chains can deposit in the glomerular mesangium and capillary loops to also cause nephrotic syndrome and renal failure in MM [3]. Infection due to immune paresis is a common cause of death in MM, and hypogammaglobulinemia due to nephrotic syndrome further increases the risk of infection. In contrast, the proposed mechanisms for COVID-19 mediated renal injury are quite varied, ranging from dehydration associated AKI to ATN associated with rhabdomyolysis/microangiopathy. Su et al. stated that LM findings in COVID-19 patients ranged from mild-to-severe acute tubular injury to mild-to-moderate arteriosclerosis [13]. EM demonstrated spherical virus-like particles characteristic of coronavirus in the proximal tubular epithelium [13].

Hence, this subset of patients may be more vulnerable to COVID-19 in the current wave. In regard to COVID-19 and cancer, there are cumulative data to indicate that patients with cancer, in particular hematological cancers, may be at an increased risk of more severe COVID-19, including those receiving or not receiving treatment within the month prior to infection [14]. It is yet unclear whether the increased risk is associated with the malignancy or treatment strategies. From a nationwide analysis in China, 18 out of 1,590 COVID-19 cases had a history of cancer. These patients were older, had more frequent abnormal computed tomography (CT) scans, and were more likely to experience an adverse event defined as admission to the ICU requiring ventilation or death [15].

In terms of treatment, the proteasome inhibitor bortezomib, a chemotherapeutic agent, cyclophosphamide, and a steroid, dexamethasone, were used in combination in this patient to treat newly diagnosed MM in the setting of COVID-19. Dexamethasone is typically used with chemotherapy to reduce light chain load in patients with MM and AKI [16]. CyBorD is an approved combination for the initial therapy of MM [17]. Plasmapheresis has also been shown to decrease light chains and improve kidney recovery [18]. A comprehensive review by Al Saleh et al. recommends bortezomib to be given subcutaneously once a week and the dose of dexamethasone reduced to 20 mg in COVID-19 patients [19]. A reduction in steroid doses in older patients and possibly an interruption of steroids in patients already in complete remission while receiving continuous treatment should be considered [19]. This treatment regimen saw no disease flares of COVID-19 in this patient and the symptoms of MM were controlled. Susek et al. observed that a cohort of patients with COVID-19 and progressive MM receiving daratumumab or in remission under lenalidomide treatment may need a closer clinical follow‐up, which they directly attributed to the treatment [20].

## Conclusions

Overall, the management of patients with MM is likely to be challenging during the current COVID-19 pandemic. MM has variable mortality for hospitalized MM patients and should be in the differential with patients who present with nephrotic syndrome and COVID-19 infection.
